# Anterior superior pancreaticoduodenal artery pseudoaneurysm after distal pancreatectomy with en bloc celiac axis resection successfully treated with balloon-assisted coil embolization

**DOI:** 10.1007/s12328-022-01710-9

**Published:** 2022-10-08

**Authors:** Shinya Ida, Yoshifumi Morita, Ryuta Muraki, Satoru Furuhashi, Makoto Takeda, Hirotoshi Kikuchi, Yoshihiro Hiramatsu, Yukichi Tanahashi, Satoshi Goshima, Hiroya Takeuchi

**Affiliations:** 1 Department of Surgery, Hamamatsu University School of Medicine, 1-20-1 Handayama, Higashi-ku, Hamamatsu, 431-3192 Japan; 2 Department of Perioperative Functioning Care and Support, Hamamatsu University School of Medicine, Hamamatsu, Japan; 3 Department of Radiology, Hamamatsu University School of Medicine, Hamamatsu, Japan

**Keywords:** Balloon-assisted coil embolization, Distal pancreatectomy with en bloc celiac axis resection, Intra-abdominal hemorrhage, Postoperative pancreatic fistula

## Abstract

Bleeding is a fatal complication after pancreatectomy. Although coil embolization is a widely accepted treatment option, ischemia of the remaining organs should be prevented. This study reports the successful treatment of intra-abdominal hemorrhage following distal pancreatectomy with en bloc celiac axis resection (DP-CAR) using balloon-assisted coil embolization (BACE). A 59-year-old man was diagnosed with locally advanced pancreatic cancer. The tumor involves the common hepatic artery, splenic artery, and celiac artery. After four cycles of treatment with gemcitabine/nab-paclitaxel, the soft-density masses, surrounding the artery, shrunk. DP-CAR and R0 resections were performed. A minor postoperative pancreatic fistula occurred. Six months postoperatively, the computed tomography showed delayed asymptomatic bleeding from an anterior superior pancreaticoduodenal artery (ASPDA) pseudoaneurysm located near the gastroduodenal artery confluence. BACE was performed by placing a microballoon catheter in the region of confluence of the ASPDA and posterior superior pancreaticoduodenal artery (PSPDA) to prevent coil migration. After inserting the microballoon catheter, coil embolization was performed in the ASPDA. Hepatic blood flow was maintained from the PSPDA. BACE is a useful technique to preserve blood flow to the remnant organs when performing coil embolization for bleeding following a distal pancreatectomy, especially following a DP-CAR.

## Introduction

The mortality rate among patients, undergoing pancreatectomy, has decreased to less than 5% due to the advances in surgical procedures and postoperative management. The complications of a pancreatectomy include postoperative pancreatic fistula (POPF) formation, delayed gastric emptying, and post-pancreatectomy hemorrhage (PPH). PPH is a fatal complication that often requires immediate treatment [1–3]. PPH occurs in 7.5–10% of patients who undergo surgery, and the mortality rate for patients with PPH was reportedly 16–50% [4, 5]. Bleeding within 24 h after surgery is considered early bleeding, and bleeding after 24 h postoperatively is considered late bleeding. Early bleeding is often caused by the technical failure of appropriate hemostasis during the operation. In contrast, late bleeding is mainly caused by POPF, intra-abdominal abscess, and bile leak [6, 7].

Laparotomy is the treatment of choice for early bleeding, while coil embolization is used to address late bleeding [8, 9]. When performing coil embolization in patients, who underwent distal pancreatectomy with en bloc celiac axis resection (DP-CAR), the hepatic arterial flow from the superior mesenteric artery (SMA) must be maintained. The disruption of the hepatic arterial flow causes liver failure and liver abscess formation [10, 11].

Balloon-assisted coil embolization (BACE) utilizes a balloon stent when performing coil embolization [12, 13]. BACE is effective for selective embolization of the anterior (ASPDA) or the posterior superior pancreaticoduodenal artery (PSPDA). It prevents the migration of the coil to another artery. This study presents a case of late-onset intra-abdominal hemorrhage, following DP-CAR, that was successfully treated with BACE.

## Case report

**Fig. 1:**
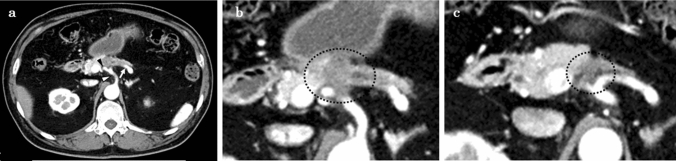
Enhanced computed tomography findings of the primary tumor. **a**: Enhanced CT showed the tumor involving the CHA (black arrowhead), SA (white arrow), and CA (white arrowhead) at the first visit. **b**: Magnified enhanced CT at the first visit showed that the tumor (black dotted circle), 24 mm in diameter. **c**: After four cycles of chemotherapy, enhanced CT showed that the tumor size had decreased 16 mm in diameter, and the soft-density mass (black dotted circle) around the CHA, SA, and CA had shrunk. *CA* celiac artery, *CHA* common hepatic artery, *CT* computed tomography, *SA* splenic artery

The patient was a 59-year-old diabetic male with gradually increasing blood sugar levels. Computed tomography (CT) revealed a pancreatic body tumor, measuring 24 mm in diameter. It involved the common hepatic artery (CHA), splenic artery (SA), and celiac artery (CA) (Fig. 1a, b). The patient was referred to our department for further treatment. We performed endoscopic ultrasound guided fine needle aspiration (EUS-FNA) for diagnosis. The biopsy identified an adenocarcinoma, and the patient was diagnosed with locally advanced pancreatic cancer. We decided to perform induction chemotherapy initially and switch to surgery if the tumor shrinkage was obtained. The patient received induction chemotherapy with gemcitabine/nab-paclitaxel (GnP). After four cycles of chemotherapy, a CT scan revealed that the soft-density mass, surrounding the CHA, SA, and CA, partially shrunk (Fig. 1c). The elevated serum tumor markers also decreased after chemotherapy as follows: CEA level was 4.6–3.5 ng/mL, CA19-9 was 86–36 U/mL, DUPAN-2 was 57 U/mL to less than 25 U/mL, and Span-1 was 42–19 U/mL. The tumor was considered resectable via DP-CAR. Blood flow to the proper hepatic artery via the gastroduodenal artery (GDA) was secured under CHA clamping. The CA was ligated twice and dissected approximately 2 cm from the root. The operation time was 379 min, and the intraoperative bleeding volume was 1,292 mL. We started administration of somatostatin analogue on the day of surgery. Three days after surgery, the patient had an elevated inflammatory reaction and CT recognized fluid collection around the pancreatic stump. We diagnosed pancreatic fistula [International Study Group of Pancreas Fistula (ISGPF)] grade BL and changed the antibiotic from cefotiam to meropenem. The drain culture was negative and the tip of the drain was away from fluid collection, so we removed the drain five days after surgery. The somatostatin analog was stopped seven days after surgery and meropenem was stopped ten days after surgery because the inflammatory reaction improved. The length of postoperative hospital stay was 19 days. The pathological diagnosis was a moderately to poorly differentiated adenocarcinoma, T1cN1aM0 Stage IIB according to the 7th edition of classification of pancreatic carcinoma from Japan Pancreas Society. The effect of chemotherapy was grade 2 [14].

**Fig. 2:**
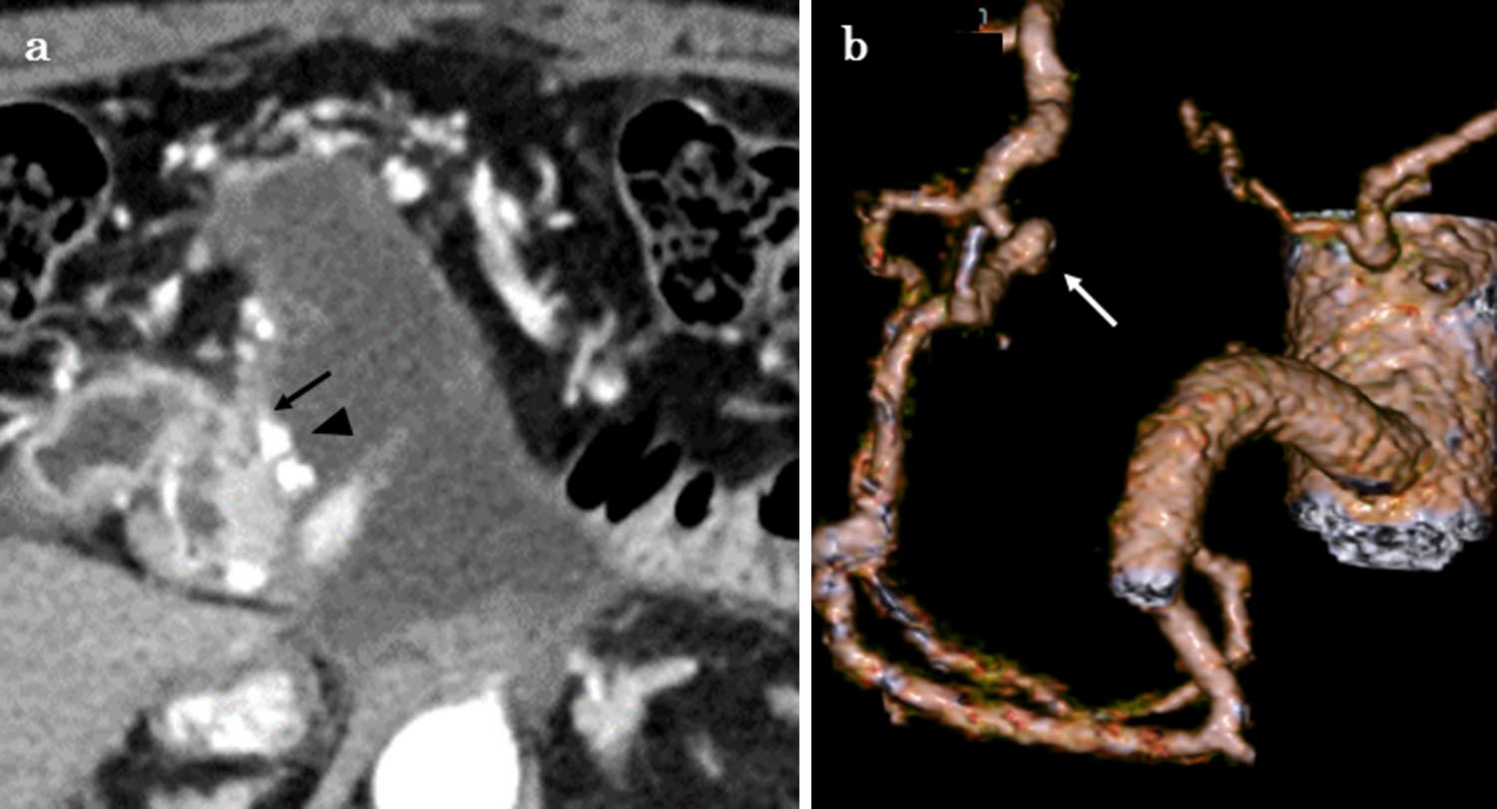
Enhanced CT findings three months after surgery. **a**: Enhanced CT image revealed a pseudoaneurysm (black arrowhead), 3 mm in diameter, arising from ASPDA (black arrow). Fluid collection was also observed near the pancreatic stump. **b**: Three-dimensional reconstruction image of the blood vessels revealed an aneurysm in the ASPDA (white arrow). *ASPDA* anterior superior pancreaticoduodenal artery, *CT* computed tomography

**Fig. 3:**
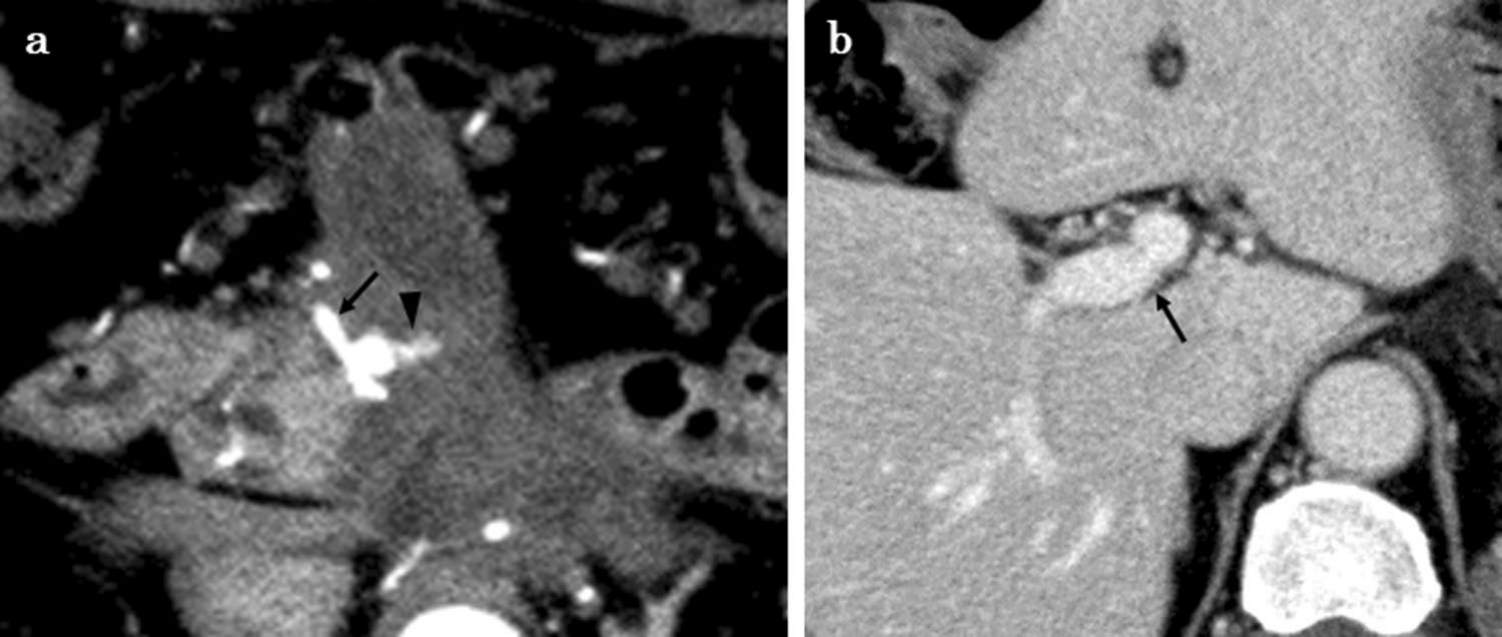
Enhanced CT findings six months after surgery. **a**: Enhanced CT showed a pseudoaneurysm arising from the ASPDA (black arrow) and asymptomatic active bleeding arising from the ASPDA pseudoaneurysm (black arrowhead). **b**: Enhanced CT showed that the blood flow of the PV was maintained (black arrow). *ASPDA* anterior superior pancreaticoduodenal artery, *CT* computed tomography, *PV* portal vein

**Fig. 4:**
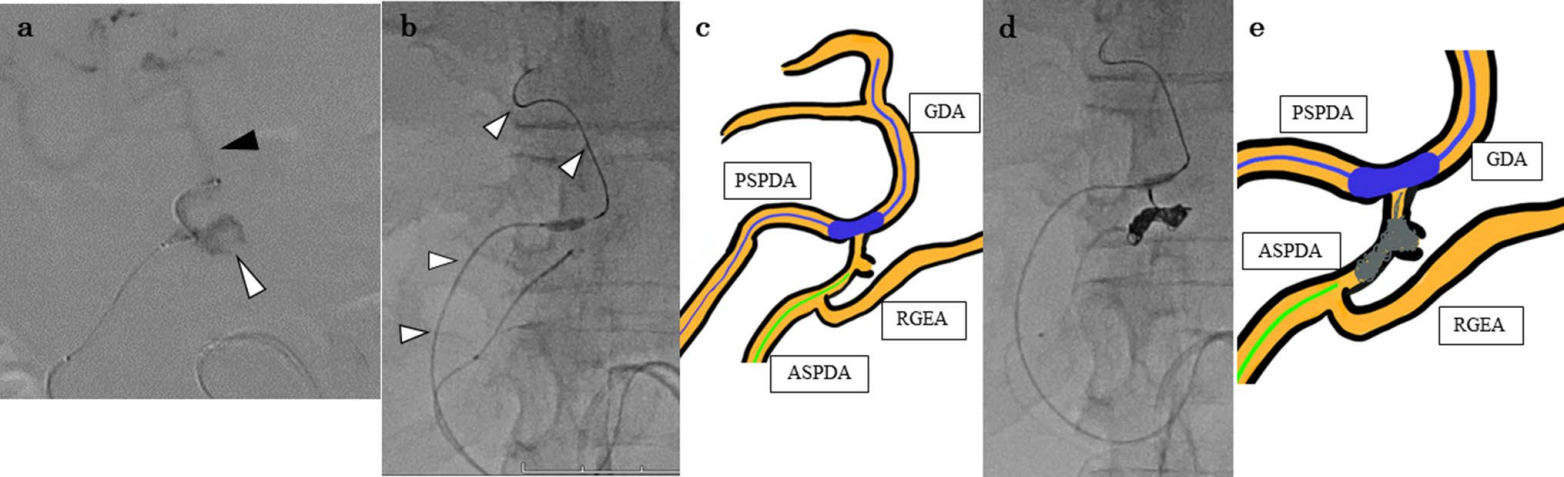
Angiography and schema during coil embolization. **a**: Angiography revealed active bleeding (white arrowhead) arising from the ASPDA pseudoaneurysm. The pseudoaneurysm was located near the root of the GDA (black arrowhead). **b**, **c**: A balloon catheter was placed via the PSPDA (white arrowhead). The confluence of the ASPDA and GDA was occluded using a balloon. **d**, **e**: The coil was placed in the ASPDA with an occluding balloon placed in the PSPDA to the GDA. *ASPDA* anterior superior pancreaticoduodenal artery, *GDA* gastroduodenal artery, *PSPDA* posterior superior pancreaticoduodenal artery

**Fig. 5:**
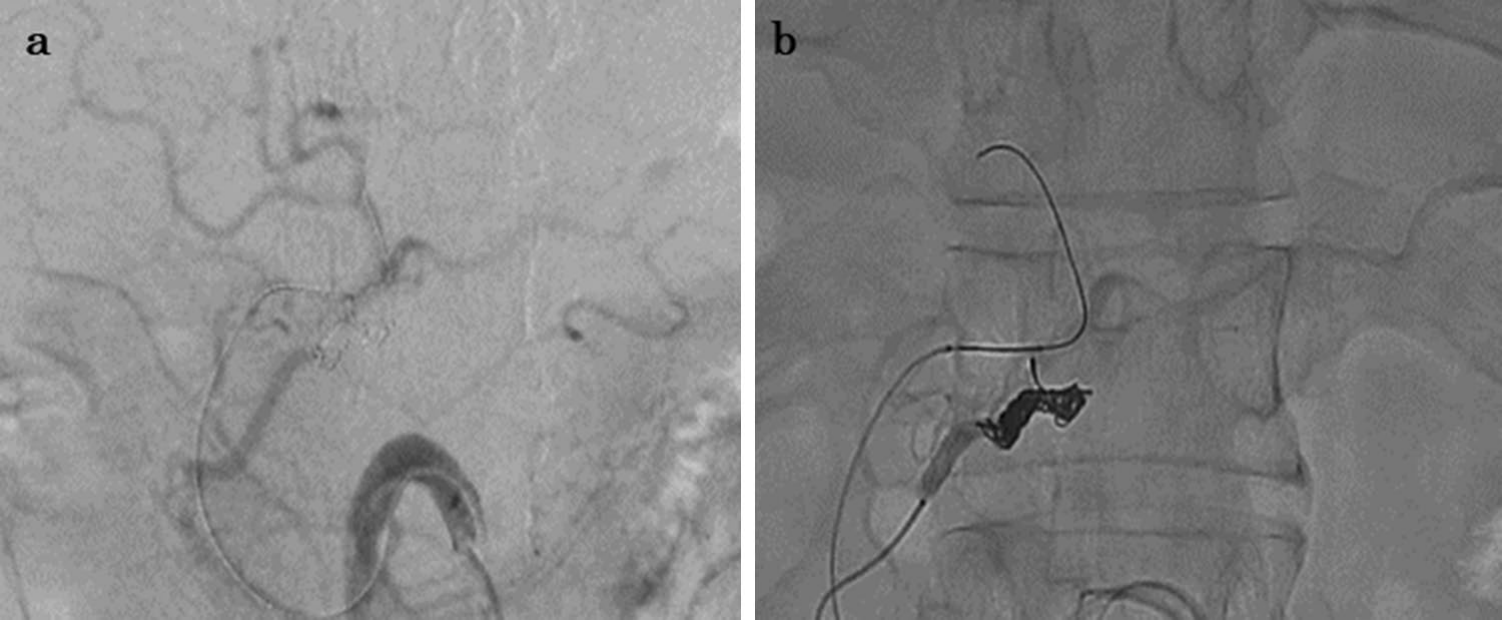
Angiography after the coil embolization. **a**: Angiography after the coil embolization through the PSPDA confirmed hepatic arterial flow. **b**: Angiography after the coil embolization of the ASPDA confirmed no active bleeding. *ASPDA* anterior superior pancreaticoduodenal artery, *PSPDA* posterior superior pancreaticoduodenal artery

Two months postoperatively, the patient received adjuvant chemotherapy with GnP. Since we confirmed that GnP was effective in this patient during induction chemotherapy, we selected GnP as postoperative adjuvant chemotherapy. Three months postoperatively, CT showed a 3 mm pseudoaneurysm, arising from the ASPDA. The pseudoaneurysm was proximal to the area of confluence of the ASPDA and GDA. Fluid collection was also observed proximal to the pancreatic stump (Fig. 2). Although the patient had received two courses of GnP, chemotherapy was discontinued at this time. Trans-gastric drainage of the fluid and angiography was initially planned. However, CT showed spontaneous reduction of the fluid collection and partial shrinkage of the pseudoaneurysm when the patient admitted to our hospital. Therefore, the patient underwent close follow-up without treatment. Three months later, enhanced CT revealed asymptomatic bleeding, arising from the ASPDA pseudoaneurysm (Fig. 3a). The blood flow of the portal vein (PV) was maintained (Fig. 3b). Since the patient had stable vital signs, coil embolization was scheduled. Migration of the coil to the GDA results in hepatic infarction and liver failure. Therefore, BACE was performed by inserting a microballoon catheter in the area of confluence of the ASPDA and PSPDA to prevent coil migration. First, both femoral arteries were punctured, and 5-Fr long sheaths were placed. The SMA was visualized using a catheter, inserted through the left sheath, to confirm the bifurcation of the pancreatic duodenal arcade and the location of the pseudoaneurysm (Fig. 4a). A microballoon catheter (Attendant SP^®^, Terumo, Tokyo, Japan) was inserted through the right sheath to the region of confluence of the ASPDA and PSPDA (Fig. 4b, c). After microballoon placement, coil embolization was performed in the ASPDA from the microcatheter (Progreat β^®^, Terumo, Tokyo, Japan), inserted through the left sheath (Fig. 4d, e). Adequate blood flow to the liver was maintained after coil embolization (Fig. 5a). Before finishing the procedure, we confirmed the absence of bleeding (Fig. 5b). Magnetic resonance imaging showed no bleeding or coil migration one month after coil embolization.

## Discussion

PPH is a fatal complication of distal pancreatectomy. Occurring in 7.5–10% of patients, it has a mortality rate of 16–50% [5, 11, 15]. The severity of bleeding following a pancreatectomy was defined by the ISGPF. It is categorized based on the time of bleeding, bleeding volume, general condition of the patient, and necessity of treatment. Bleeding within 24 h postoperatively is defined as early bleeding [8, 16, 17]. In terms of bleeding volume, a decrease in hemoglobin of less than 3 mg/dl is defined as mild, while a decrease in Hb of 3 mg/dl or more is defined as severe. Grade A refers to early and mild bleeding, that is treated conservatively. Meanwhile, Grade B refers to early but severe bleeding, or mild but late bleeding. Lastly, Grade C refers to late and severe bleeding. Early treatment is important because hemorrhagic shock and coagulopathy have a poor prognosis [4, 10, 18]. Grades B and C require invasive treatments, including endovascular treatment and laparotomy [16, 17, 19]. In the present case, an ASPDA pseudoaneurysm was observed three months postoperatively. At that time, there was no bleeding from the pseudoaneurysm and no complaints of abdominal symptoms from the patient. We planned to hospitalize the patient for angiography and drainage of fluid collection in the pancreatic stump. However, CT at the time of admission showed spontaneous reduction of the fluid collection and partial shrinkage of the pseudoaneurysm, so we decided to follow-up in a short period of time after multi-professional team conference. Six months postoperatively, an enhanced CT showed asymptomatic bleeding from the ASPDA pseudoaneurysm, which required treatment.

Due to the advances in endovascular treatment, several cases have been treated without laparotomy [2, 20, 21]. In a study comparing endovascular treatment and laparotomy, the complication and mortality rates of endovascular treatment were lower than those of laparotomy [9, 19, 22]. Endovascular treatment is particularly useful for managing late bleeding.

Maintaining hepatic arterial flow during endovascular treatment is associated with a reduced incidence of complications, such as liver failure and liver abscess formation [18, 20, 21]. Therefore, limited embolization of the GDA stump has been performed to address intra-abdominal bleeding following pancreaticoduodenectomy. However, the rebleeding rates remained high. Therefore, extended embolization of the hepatic arteries is recommended for patients with severe bleeding because it immediately stops the bleeding [22]. Stent grafts are also effective in patients with stable vital signs because they maintain blood flow to the peripheral organs. However, there is a specified distance, thickness, and angle of blood vessels for stent-graft placement [18, 22]. In the present case, the pseudoaneurysm was proximal to the region of confluence of the ASPDA and GDA, so the distance for stent-graft placement was inappropriate. Furthermore, the diameter of the ASPDA was too small for stent-graft placement.

In patients who underwent DP-CAR, the blood supply of the liver comes from the pancreaticoduodenal arcade through the SMA. When a pseudoaneurysm occurs in the ASPDA or PSPDA, blood flow through another vessel should be maintained to prevent reduced hepatic arterial flow. BACE prevents coil migration by temporarily occluding the artery with a balloon stent [12, 13]. Selective coil embolization of the target area reduces the incidence of complications.

BACE is suitable for patients with blood vessels that need to preserve blood flow. BACE is often used for cerebral aneurysms [23]. When the severe bleeding with unstable vital signs occurred, there is little time. Therefore, it is often necessary to widely perform coil embolization. However, skilled radiologists can perform BACE when the patient condition is stable. BACE is a safe and reliable method of coil embolization for patients that are difficult to treat via simple endovascular therapy due to anatomical variations or postoperative complications. Although this is a first reported case in which BACE was performed on pseudoaneurysm after DP-CAR, we consider that BACE is reliable and effective treatment technique. If the patient condition is stable or the procedure can be quickly completed, BACE should be considered because it can maintain blood flow to the remnant organs.

In conclusion, bleeding following a pancreatectomy is a fatal complication, often requiring immediate treatment. When performing coil embolization for bleeding from a pseudoaneurysm, it is important to maintain the blood flow to the remnant organs. BACE is a useful technique for performing selective coil embolization at the intended site while maintaining blood flow.

## Abbreviations

ASPDA: Anterior superior pancreaticoduodenal artery
BACE: Balloon-assisted coil embolization
CA: Celiac artery
CHA: Common hepatic artery
CT: Computed tomography
DP-CAR: Distal pancreatectomy with en bloc celiac axis resection
EUS-FNA: Endoscopic ultrasound guided fine needle aspiration
GnP: Gemcitabine/nab-paclitaxel
GDA: Gastroduodenal artery
ISGPF: International Study Group of Pancreatic Fistula
POPF: Postoperative pancreatic fistula
PPH: Post-pancreatectomy hemorrhage
PSPDA: Posterior superior pancreaticoduodenal artery
PV: Portal vein
SA: Splenic artery
SMA: Superior mesenteric artery
